# The role of *Akkermansia muciniphila* in human disease: a friend or a foe?

**DOI:** 10.3389/fmicb.2026.1974213

**Published:** 2026-10-30

**Authors:** Chunrong Huang, Yuqi Zhang, Hongyang Xie, Guochao Shi

**Affiliations:** 1 Department of Respiratory and Critical Care Medicine, Ruijin Hospital, Shanghai Jiao Tong University School of Medicine, Shanghai, China; 2 Institute of Respiratory Diseases, Shanghai Key Laboratory of Severe Respiratory Infection, Ruijin Hospital, Shanghai Jiao Tong University School of Medicine, Shanghai, China; 3 Department of Ultrasound Medicine, Mianyang Central Hospital, School of Medicine, University of Electronic Science and Technology of China, Mianyang, Sichuan, China; 4 Department of Cardiovascular Medicine, Ruijin Hospital, Shanghai Jiao Tong University School of Medicine, Shanghai, China

**Keywords:** *Akkermansia muciniphila*, cardiovascular, gut, metabolic disease, neurological diseases

## Abstract

*Akkermansia muciniphila* (*A. muciniphila*) was isolated and identified about two decades ago. It has drawn great attention due to its close association with diseases, such as obesity, diabetes, inflammatory bowel disease (IBD), liver steatosis, asthma and cardiovascular diseases. Research on the biological functions of *A. muciniphila* in human diseases has made it a potential probiotic with several therapeutic properties: it helps maintain gut barrier function, reduce brain atrophy, release inflammatory responses, and improve bone health. Its abundance and activity, however, are shaped by a complex interplay of environmental and host factors, including diet, medication, oxygen, bile acids, and microbial interactions. Here we review the genotypic diversity of *A. muciniphila* and its involvement in oral, atopic, respiratory, intestinal, metabolic, cardiovascular, rheumatic, skeletal, and neurological diseases, with particular attention to its bioactive components—such as the outer membrane protein Amuc_1100, extracellular vesicles, and the GLP-1-inducing protein P9—and the mechanisms through which they act. We also weigh the evidence that, in certain host contexts or in the presence of specific pathogens, *A. muciniphila* may aggravate rather than alleviate disease. A clearer, strain-level understanding of these context-dependent effects will be essential if *A. muciniphila* is to move from a promising commensal to a safe and effective therapy. The isolation, identification of *A. muciniphila* strains with beneficial effects and the large-scale industrial production challenges the translational applications.

## Background

With the initiation of the Human Microbiome Project, culture-independent methods have yielded a far more detailed picture of the microorganisms that inhabit the human body. The human gut contains up to 10^14^ bacteria (Sender et al., 2016), gut microbial dysbiosis is accompanied by the production of abnormal metabolites and dysregulation of the immune system, thereby accelerating the occurrence of intestinal, neurological, metabolic and cardiovascular diseases (Gomaa, 2020). *Akkermansia muciniphila* (*A. muciniphila*), a commensal gram-negative anaerobe, was first isolated from the feces of healthy Caucasian women in 2004. It is oval shaped and non-motile. The mucin-degrading *A. muciniphila* is present in the human gut from infancy, its relative abundance increases gradually from 1% in infancy to 4% in adulthood (Collado et al., 2012). It can colonize in cecum and small intestine, with the cecal mucosa carrying the highest load (Geerlings et al., 2018). The idea that *A. muciniphila* already existed in the intestine in early life may be explained by its detection in breast milk of human beings, which could act as a carrier of *A. muciniphila* and transfer it from mother to the infant (Collado et al., 2012; Ward et al., 2013). Despite its low abundance in the gut, *A. muciniphila* has already become a research hotspot and is regarded as a next-generation probiotic due to its anti-obesity, inflammation inhibition, and anticancer properties (Zhao et al., 2017; Chen et al., 2020; Luo et al., 2021). Many studies suggested that oral administration of live *A. muciniphila* could inhibit the pathological processes, such as fat mass gain, body weight gain, glucose intolerance in high-fat feeding mouse models (Zhao et al., 2017). In this review, we summarized the characteristics of *A. muciniphila*, the factors that shape its growth, the effect of *A. muciniphila* in various diseases as well as its underlying mechanisms.

Studies were found by searching the PubMed, MEDLINE, Scopus and Web of Science. In order to minimize the loss of studies and improve the comprehensive inclusion of studies in the present review, the following words in search strategy were used, both as free-text and topic headings: “*Akkermansia muciniphila*,” “*A. muciniphila*,” “next-generation probiotic,” “new-generation probiotic,” “Crohn disease,” “ulcerative colitis,” “inflammatory bowel disease”, “intestinal infection,” “clostridioides difficile,” “acute appendicitis,” “lung disease,” “asthma,” “dermal diseases,” “allergy,” “atopic diseases,” “eczema,” “rheumatic diseases,” “autoimmune disease,” “alcoholic liver disease, “nonalcoholic fatty liver disease”, “hepatocellular carcinoma,” “nonalcoholic steatohepatitis,” “Alzheimer’s disease”, “Parkinson’s disease”, “amyotrophic lateral sclerosis”, “Multiple sclerosis”, “encephalomyelitis”, “neurological disorders”, “stress”, “depressive”, “skeletal disorders”, “bone”, “Osteoporosis”, “oral diseases”, “Periodontitis”, “cardiovascular diseases”, “Atherosclerosis”, “hypercholesterolemia”, “obesity”, “diabetes”, “body fat”, “glucose.” The reference lists from selected studies were manually reviewed to find any other related studies. Studied published through December 2025 were included. Studies without full texts and that were not on the topic mentioned in this paper were excluded.

## Genotypic diversity of *A. muciniphila*

Kirmiz et al. conducted a pangenomic analysis of 35 *A. muciniphila* genomes from human beings and identified four phylogroups (clades AmI–AmIV) with different functions. For instance, genes in the AmII phylogroup potentiate vitamin B_12_ biosynthesis, thereby triggering the production of propionate (Kirmiz et al., 2020). *A. muciniphila* MucT lacks the genes encoding synthetase of the corrin ring of vitamin B12, a cofactor required for nucleotide synthesis and methionine synthesis, and for propionate metabolism in this bacterium (Shelton et al., 2019). Propionate regulates appetite through stimulating human colonic cells to release peptide YY (PYY) and glucagon-like peptide-1 (GLP-1) (Chambers et al., 2015). In 2015, Guo et al. isolated 22 *A. muciniphila* strains from human fecal samples and identified 12 subtypes using DNA fingerprint analysis (Guo et al., 2016). They further analyzed 39 *A. muciniphila* genomes from mouse and human feces and identified three species of *A. muciniphila* possessing different metabolic functions and features (AmI, AmII, and AmIII) using phylogenetic analysis. The proteins unique to each phylogroup had distinct functional profiles. For instance, the AmI phylogroup was enriched for the phosphatidylinositol signaling system, degradation of aromatic compounds, ATP-binding cassette (ABC) transporters. The phosphatidylinositol signaling system comprises the enzymatic machinery that phosphorylates phosphatidylinositol to generate phosphorylated phosphoinositides and diacylglycerol, molecules that serve as intracellular messengers and participate in the remodeling of membrane phospholipids. In bacteria, this category reflects a distinct capacity for membrane lipid metabolism, whose relationships with *A. muciniphila* remain to be defined, although this bacterium is known to remodel its membrane lipids when challenged by bile acids (Hagi et al., 2020). The degradation of aromatic compounds refers to the enzymatic breakdown of ring-structured molecules, a large share of which in the gut are dietary polyphenols. Polyphenol-rich diets reproducibly increase the intestinal abundance of *A. muciniphila*, most notably in mice fed a cranberry extract (Anhê et al., 2015), suggesting that the capacity to handle aromatic compounds may help this bacterium thrive under polyphenol exposure. ABC transporters constitute one of the largest superfamilies of bacterial membrane proteins and use the energy of ATP hydrolysis to move substrates across the cell membrane, importing nutrients and exporting metabolic by-products and toxic compounds. In *A. muciniphila*, gene clusters encoding an ABC transporter can be regulated by bile acids and their pharmacological inhibition of ABC transporter reduces bile acid tolerance of *A. muciniphila* (Hagi et al., 2020). However, the AmII/AmIII phylogroups harbored more proteins involved in amino sugar and nucleotide sugar metabolism, sulfur relay system, fructose and mannose metabolism. Amino sugar and nucleotide sugar metabolism covers the processing of amino sugars such as N-acetylglucosamine and *N-acetylgalactosamine* and their activated nucleotide-sugar forms, which are central to the living of *A. muciniphila*, because they are the building blocks of mucin glycans and *N-acetylglucosamine* is a prerequisite for the growth of *A. muciniphila* (van der Ark et al., 2018). Fructose and mannose metabolism describes the utilization of hexoses, which reach the gut both from dietary fibers and from host glycans. The increase of *A. muciniphila* abundance was observed in obese mice fed the prebiotic oligofructose. Both AmII and AmIII genomes are enriched for carbohydrate-active enzymes (CAZymes) which can decompose dietary polysaccharides and carbohydrates more efficiently (Guo et al., 2017), thus far, affecting metabolic derivatives and their interactions with the host immune system. Recently, Becken et al. reported four *A. muciniphila* phylogenetic groups from 71 strains isolated from adolescents and children, these groups differed in oxygen tolerance, TLRs activation, the ratio of acetate/propionate fermentation end products, and sulfur acquisition that likely affected their growth, intestinal colonization, immune responses and metabolic processes of the host (Becken et al., 2021). Interestingly, one *A. muciniphila* phylogroup may predominate at a certain time and its abundance turn into another over time in an individual (Becken et al., 2021). However, how they differed in their ability to influence human health is largely unknown and delineating phylogenetic-specific traits warrants further study.

Although two genomes showed high similarity (>99%), different *A. muciniphila* strains may differ in biological functions, such as metabolism of dietary components or the generation of anti-inflammatory metabolites. Indeed, some strains are demonstrated to be beneficial, whereas others are neutral or potentially harmful (Cirstea et al., 2018). Moreover, strains that are beneficial in one host context may become harmful in another, depending on the disease status (Seregin et al., 2017). Thus, it would be more persuasive to stratify the most abundant *A. muciniphila* phylogroup in an individual when illustrating the observed associations between *A. muciniphila* and diseases. Isolating strains, identifying the most beneficial ones, characterizing the metabolites they produce and their biological functions, and clarifying the determinants of strain-specific colonization will all be important for advancing the development of effective *A. muciniphila*-based probiotics. Here, we summarized the effects of *A. muciniphila* on diseases (Table 1).

**Table 1:** Functions of different strains.

Origin	Strains	Function	Target
Human	Akk ATCC BAA-835	augment Treg differentiation	Intestine
		ease inflammation and apoptosis of TNF-α-stimulated IECs	Intestine
		relieve inflammation severity and infiltration by activating colon NLRP3 in DSS-induced acute colitis	Intestine
		alleviate colitis by inhibiting macrophage infiltration and CD8 + CTLs	Intestine
		restore the reduced secondary BAs levels in CDI mice, relieve colonic inflammation by restraining TLR4/MyD88 and mTOR signaling pathways in the gut, and protect the mucosal immune barrier	Intestine
		Protect against S. pullorum infection in the intestine by stimulating the proliferation of intestinal stem cells through the Wnt/β-catenin signaling pathway	Intestine
		accelerate gut inflammation in *Salmonella Typhimurium*-infected gnotobiotic mice, such as increasing histopathology scores, promoting macrophage infiltration in cecal tissue, and elevating inflammatory cytokines in cecal and colonic tissues	Intestine
		hamper H7N9 virus proliferation, improve pathologic changes in the lung and clinical symptoms, and inhibit cytokine release in the lungs during H7N9 infection	Lung
		decrease mortality and alleviate weight loss in mice infected with the PR8 influenza virus	Lung
		increase in ankle swelling in a mouse model of arthritis	Rheumatic diseases
		facilitate l-aspartate transportation from the gut to the liver, which further stimulated LKB1 AMPK axis activation and lipid oxidation, thus relieving MAFLD in mice	Liver
		regulate TG synthesis in the liver and maintain gut homeostasis	Liver
		reduce infiltration of neutrophils and macrophages in an immune-mediated liver injury mouse model	Liver
		reduce fibrosis markers (e.g., TGF-β, α-SMA, PDGF, TLR-2, and TIMP), lighten inflammation, and normalize gut microbiome in HFD/CCl4-induced liver injury	Liver
		improve motor function, decrease brain atrophy, and prolong the lifespan of ALS animal models	Neuron
		induce mitochondrial calcium overload and α-synuclein aggregation in an enteroendocrine cell line	Neuron
		improve cognitive dysfunction, anxiety-related behaviors, and amyloid pathology in a mouse model of AD	Neuron
		modulate gut microbiota and metabolites, affect hormone levels, and promote the synaptic signaling pathway and neuronal connections in a mouse model of depression induced by CRS	Neuron
		relieve the EAE model of MS by inducing Tregs in a mechanism dependent on increased TGF-β and reduced IL-1β and IL-6 release by DCs	Neuron
		restrain the inflammatory response at the early stage of fracture site and trigger type H vessel formation in callus and angiogenesis, thus promoting fracture healing and stimulating bone formation	Bone
		restore the OVX-induced imbalanced bone metabolism and protect against osteoporosis	Bone
		decrease inflammation of periodontal tissue and calvarial bone destruction, improve the expression of tight junction and cell–cell adhesion markers, and promote IL-8 expression in gingival epithelial cells, ultimately decreasing bone loss caused by P gingivalis inoculation	Bone
		induce beneficial M2 macrophages in submandibular lymph nodes and augment IL-10 levels in gingival tissues and macrophages, thus mitigating P gingivalis-induced alveolar bone loss	Oral
		reduce fat mass, hyperglycemia, glucose intolerance, IR, and dyslipidemia in obese and diabetic mice	metabolic diseases
		alleviate metabolic endotoxemia, activate insulin signaling in the liver and muscle, and improving global glucose tolerance	metabolic diseases
		reduce hepatic glycogen levels and elevate HDL-C levels, enhancing resistance to gluco/lipotoxicity	metabolic diseases
		reduce proteins representing ER stress, counteract oxidative damage	metabolic diseases
		promote GLP-1 secretion, inhibite β-cell apoptosis, stimulate β-cell replication, neogenesis, and differentiation	metabolic diseases
		increase mucus production and expression of the antimicrobial peptide Reg3γ, lower serum endotoxin levels and islet TLR expression, reduce TLR2/4 expression and increase Foxp3 + regulatory T cells in islets, increase IL-10 and TGF-β in pancreatic lymph nodes, thus regulating immune responses and inhibiting T1D development	metabolic diseases
		reduce the OGTT peak, prevent the increase in intestinal absorptive capacity, reverse the decrease in apoptotic cells, and reduce glucose uptake genes induced by cold exposure	metabolic diseases
Human	Akk strains (EB-AMDK-10, 19, 27)	decrease body weight gain, fat mass, calorie intake, adipogenesis/lipogenesis, serum total cholesterol (TC) levels, and inflammatory insults	metabolic diseases
Human	Akk strain 139	Fail to augment Treg differentiation	/
Human	Akk FSDLZ36M5	protect against UC	Intestine
Human	FSDLZ-39M14, 20M4	fail to protect against UC	Intestine
Human	Akk FSDLZ20M4	accelerate colon inflammation	Intestine
Human	Akk EB-AMDK19	ameliorate dermatitis score, scratching behavior, and serum IgE levels; decrease TSLP and Th2 cytokine levels; prohibit infiltration of mast cells and eosinophils in skin lesions; and relieve colonic damage in DNCB-induced AD models	Skin

## Factors influencing the growth and metabolites of *A. muciniphila*

### Environmental factors

The growth of *A. muciniphila* can be affected by many factors, such as oxygen levels, intestinal pH, and bile acids (Flint et al., 2012). Oxygen liberated by epithelial cells cause serious damage to anaerobic gut bacteria. *A. muciniphila* impedes the harm of oxygen and survives in the gut through an oxygen consumption system that depends on the cytochrome bd complex (Ouwerkerk et al., 2016). However, Ouwerkerk et al. measured the oxygen tolerance and reported that *A. muciniphila* could survive in ambient air for up to 48 h; the viability descended to 25% after 24 h incubation and to only 1% after 48 h (Ouwerkerk et al., 2016). Machado et al. reported that *A. muciniphila* showed a high oxygen tolerance at room temperature and refrigeration for up to 72 h, challenging the view that oxygen causes damage to *A. muciniphila* (Ouwerkerk et al., 2016). Simultaneously, *A. muciniphila* showed culture stability at room temperatures and refrigeration (Machado et al., 2020). Detailed information addressing the effect of environmental factors (eg. aerobic atmosphere or temperature) on viability of *A. muciniphila* is critital for the utilization of *A. muciniphila* as a next-generation probiotic candidate. In addition, other studies have also indicated the susceptibility of *A. muciniphila* to environmental pollutants, such as benzopyrene and heavy metals (Ribière et al., 2016; Zhai et al., 2017; Li et al., 2019b). Cadmium (Cd) exposure, for instance, significantly reduced the relative abundance of *A. muciniphila* in the mouse gut (Feng et al., 2019).

### Medications

Medications also shape the growth of *A. muciniphila*. Metformin, a type 2 diabetes treatment, exert anti-inflammatory and mucus barrier protective effects in mice with ulcerative colitis (UC) by enriching intestinal *A. muciniphila* (Ke et al., 2021). The pomegranate peel, which accounts for roughly 30-40% of the fruit, is an important component used in traditional medicine due to its anti-inflammatory, antioxidant, antidiabetic, anticancer, and antihyperlipidemic activities. Pomegranate peel improved gut microbial dysbiosis in diabetic retinopathy rats, it particularly increases the abundance of *A. muciniphila* (Ren et al., 2026). Berberine is a traditional Chinese medicine, shows promise in treating Metabolic dysfunction-associated steatohepatitis (MASH) partially through facilitating *A. muciniphila* colonization (Xu et al., 2025). Si-Ni-San is therapeutic in colitis dependent on gut microbiota, and it particularly favors the growth of *Akkermansia* genus (Cai et al., 2023). Fujisaka et al. also identified several herbal drug extracts that significantly increased *A. muciniphila* DNA levels, among them, dioscoreae rhizoma extract was demonstrated to increase the gut *A. muciniphila* growth in mice fed with a high-fat diet and improve obesity (Fujisaka et al., 2024).

### Dietary patterns

A growing number of studies have explored the relationship between diet and human diseases, which are often accompanied by fluctuations in the gut microbiota. Fibers, meat, proteins and other bioactive components in food are closely associated with changes in the gut microbiota. Zhao et al. investigated the effect of different dietary proteins on the gut microbiota and human health. Their data showed that a soy-protein-based diet reduced the ratio of *A. muciniphila*, mucus layer thickness, goblet cell number, and mRNA levels of Muc2 in the colon of mice, while a chicken protein-based diet statistically increased the proportion of *A. muciniphila* in the gut (Zhao et al., 2019). Further work implied that the two types of protein-based diets altered *A. muciniphila* genes involved in energy metabolism, such as oxidative phosphorylation and ribosome function (Zhao et al., 2019). On the other hand, a high-salt diet was demonstrated to be intimately associated with hypertension, cardiovascular diseases and obesity, moreover the gut microbiota emerged as an important “middle effector” (Erdem et al., 2010; Gao et al., 2022; Lanaspa et al., 2018). Studies showed that high fecal salinity decreased *A. muciniphila*, *Bifidobacterium longum*, and *Bifidobacterium adolescent*, organisms with anti-obesity roles that are regarded as key symbionts for metabolic and immune processes (Schneeberger et al., 2015; Everard et al., 2013; Tan et al., 2016; Schell et al., 2002). Therefore, these data may provide more therapeutic options for obesity in the future. In addition, Various dietary compounds—including extracts of dark sweet cherry, lingonberry, alpine bearberry, and cloudberry, fucoidan, inulin, polysaccharides from *Gastrodia elata*, tryptophan, and *Ophiopogon japonicus*—have been shown to significantly increase the growth of *A. muciniphila* (Garcia-Mazcorro et al., 2018; Anhê et al., 2018; Guo et al., 2021; Huo et al., 2021; Zhang et al., 2022). Green tea is a representative oriental tea with anti-inflammatory, antidiabetic, anti-obesity, and anticancer properties (Shin et al., 2018; Kim et al., 2010; Cunha et al., 2013). Green tea has been shown to release obesity and promote the growth of *A. muciniphila* in diet-induced obese mice (Jeong et al., 2020). Pomegranate ellagitannins can also promote *A. muciniphila* growth *in vivo* (Henning et al., 2017).

### Host factors

Lifestyle and other organisms also influence the proportion of *A. muciniphila*. For instance, co-culture with the butyrogenic commensal intestinal *Anaerostipes caccae* and exercise led to increased abundance of *A. muciniphila* (Bressa et al., 2017; Chia et al., 2018). Zhang et al. compared the fluctuation of *A. muciniphila* and other beneficial bacteria in the gut between healthy controls and patients with inflammatory bowel disease (IBD), they found that the proportion of *A. muciniphila* exhibited a positively correlation with that of *Faecalibacterium prausnitzii* (*F. prausnitzii*) in controls (Zhang et al., 2020b). A plausible reason is that *A. muciniphila* releases oligosaccharides and then produces acetate through degrading mucus and fermenting, which was utilized by microbes near the intestinal mucosa, such as *F. prausnitzii*, to promote its growth. Therefore, incubation of *A. muciniphila* with *F. prausnitzii* led to mutual growth (Belzer et al., 2017). In addition, *A. muciniphila* colonized in the gut, it cannot survive in an intestinal environment with pH below 5.5 (Derrien et al., 2004). Besides, *A. muciniphila* was exposed to bile acids. It has been showed that bile salts of human beings affected the growth of *A. muciniphila*, such as the inhibitory effect of ox-bile and the enhanced effect of sodium deoxycholate. A 0.1% bile salt mixture statistically increased the growth of *A. muciniphila* (Hagi et al., 2020).

The above discussion about the associations between medications, intestinal pH, oxygen, diet source or ingredients, lifestyle, microbial interactions, and the growth of *A. muciniphila* suggests the potential mechanisms of the effect of medications, environmental factors, diet and lifestyle on human health and underscores the paramount involvement of *A. muciniphila* in human disease (Figure 1).

**Figure 1:**
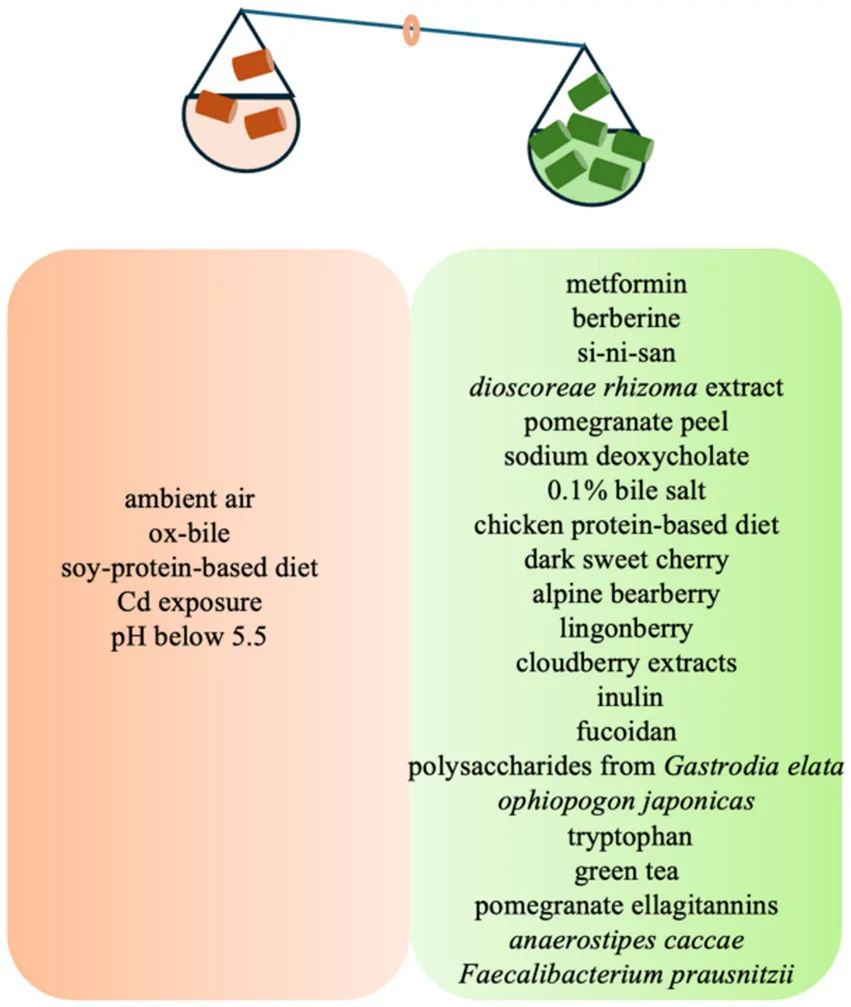
Factors influencing the growth of *A. muciniphila*.

#### *A. muciniphila* and intestinal disease

*IBD* is a chronic inflammatory disease of in the gut that consists of Crohn disease (CD) and UC. It is commonly characterized by gut microbial dysbiosis, dysfunction of intestinal barrier, and immune system disorder, and its rising incidence imposes a heavy burden on human health (Kaplan and Windsor, 2021).

##### Human studies

Patients with IBD exhibited a decreased proportion of *A. muciniphila* in the gut (Lo Presti et al., 2019)(Lo Sasso et al., 2021; Kump et al., 2018; Lopez-Siles et al., 2018; Zhang et al., 2020b). These data point to a striking association between the decline of *A. muciniphila* abundance and IBD, and dysbiosis of *A. muciniphila* has been regarded as a potential therapeutic target for IBD prevention. However, Danilova et al. reported partially contradictory results: the proportion of *A. muciniphila* was significantly increased in patients with CD and decreased in patients with UC (Danilova et al., 2019). It’s worth to note that the CD patients in Danilova’s research contains rural and urban residents, indicating that this conflicting conclusion about the abundance of *A. muciniphila* in CD may be explained by the sample population and the living environment. In addition, CD can affect any part of the digestive tract, and different segments harbor distinct dominant microbiotas. Most human studies, however, have not distinguished the gut microbiota of CD patients according to the location of the main lesions, such as the ileum or colon, which may further explain the controversy.

##### Animal and *in vitro* studies

In animal studies, the controversial results about the abundance of gut *A. muciniphila* also existed. Many studies showed a decrease in the abundance of *A. muciniphila* in mice models of colitis, relative to the controls (Alvarado et al., 2019; Yang et al., 2018). However, there were also reports stated that the ratio of *A. muciniphila* in the gut was significantly augmented in mice with colitis (Håkansson et al., 2015; Zhang et al., 2016). The conflicting results may be explained by multiple factors such as the different animal models used. The abundance of *A. muciniphila* in IBD therefore remains contentious; even though, we cannot deny the vital role of *A. muciniphila* in regulating the gut immune system, which has been confirmed by growing evidence.

*A. muciniphila* have been shown to possess strain-specific regulation of human immunity in UC. Zhai et al. reported that both *A. muciniphila* type strain ATCC BAA-835 and strain 139 isolated from mice showed anti-inflammatory effects in a mouse models of chronic colitis, although statistical significance was reached only for ATCC BAA-835. Moreover, strain ATCC BAA-835 increased the differentiation of Tregs, colonic GPR43 gene expression and short chain fatty acids (SCFAs) productions in the gut of mice with chronic colitis, whereas these effects were not found in strain 139 (Zhai et al., 2019). The differing anti-inflammatory properties of the two strains of *A. muciniphila* may lies in the strain-specific genomic characteristics that shape their different immune interactions with the host. Another explanation is the difference in how they compete with other microbes and thereby limit the colonization of pathogens in the gut. These explanations will be investigated in further studies. Liu et al. isolated three human *A. muciniphila* strains (FSDLZ-36M5, 39M14, and 20M4) and explored the effect of these strains and the *A. muciniphila*-type strain ATCC BAA-835 on dextran sulfate sodium (DSS)-induced colitis in mice. Only *A. muciniphila* FSDLZ-36M5 exhibited a protective role against UC in mice; whereas other strains (FSDLZ-20M4, 39M14 and ATCC BAA-835) did not have these effects. Interestingly, *A. muciniphila* FSDLZ20M4 tends to aggravate colon inflammation in mice (Liu et al., 2021b). The genetic difference affected the effects of *A. muciniphila* strains on colitis. Their further study showed strain-specific COG categories were detected in strain FSDLZ36M5 but not in strain FSDLZ20M4. Among these genes, gene metE is involved in methionine formation, which can promote protein synthesis and regulate the gut mucosal immune response and gut barrier (Bauchart-Thevret et al., 2009). Cpt, res, and hsdR are responsible for immune defense that can help bacterial strains develop self-defense under harsh circumstances, thus providing them a colonization advantage (Kholodii et al., 1995; Ferri et al., 2010; Morash et al., 2010). Therefore, selecting strains with particular genotypic features may be an effective method to identify strains with specific functions that could be clinically exploited. In summary, these findings suggested distinct strain-specific physiological effects on colitis in mice, the clinical applications of specific *A. muciniphila* strains are still limited by the challenges of isolation and large-scale industrial production.

Growing evidence has investigated the effect of derivatives of *A. muciniphila* on diseases. Several studies have demonstrated that gut microbiota or it-derived extracellular vesicles (EVs) showed protective roles in regulating intestinal immune responses (Shen et al., 2012; Mazmanian et al., 2008; Newburg and Walker, 2007). Kang et al. found significant changes in the composition of bacteria-derived EV (eg. EVs from *A. muciniphila*) in the large intestine in mie with colitis or UC, while only minor changes in bacterial composition were observed, indicating a striking correlation between IBD and *A. muciniphila*-derived EV (Kang et al., 2013). Moreover, *A. muciniphila*-derived EV were shown to protect against weight loss, high disease activity index (DAI)score, shortened colon length in DSS-induced colitis (Kang et al., 2013). Chelakkot et al. found that EVs derived from *A. muciniphila* improved intestinal permeability in Caco-2 cells by regulating the tight junction protein occludin through activating AMPK(Chelakkot et al., 2018). Plovier et al. also showed the effect of *A. muciniphila* on the intestinal integrity and activated cells expressing TLR2 in mice. They further found that the outer membrane protein of *A. muciniphila* (named Amuc_1100) significantly increased tight junction proteins expressions in the jejunum, including Cldn3 (encoding claudine-3) and Ocln (encoding occludin) (Plovier et al., 2017). These effects can be explained by the ability of Amuc_1100 to activate TLR2, which is known to regulate tight-junction proteins such as occludin and claudin-3 to maintain epithelial barrier function (Cario et al., 2007; Gu et al., 2016). These results indicated that *A. muciniphila*-derived EV profoundly reinforced tight junction proteins through activating AMPK pathway, while Amuc_1100 maintained the epithelial barrier through activating TLR2 and participated in the protective function of *A. muciniphila* in IBD.

Extensive studies have investigated the mechanisms underlying the effects of *A. muciniphila* on IBD. Excessive apoptosis of intestinal epithelial cells (IECs) exacerbated gut barrier dysfunction, intestinal damage and bacterial translocation (Perez et al., 2021). UC patients showed excessive apoptosis of IECs, aberrant structure and functions of the colon epithelium in colon tissue (Lin et al., 2017). Animal experiments suggested that *A. muciniphila* exerts its favorable effects by directly contacting the IECs of the host. Both active and inactive *A. muciniphila* alleviated the inflammation and excessive apoptosis of TNF-α-stimulated IECs, although the potential mechanism may be different (Luo et al., 2021): the active form reduced IEC apoptosis by downregulating key genes of the calcium signaling pathway, whereas the inactivated form reduced apoptosis by upregulating key genes of the cell-cycle signaling pathway, and both forms improved IEC inflammation by diminishing the expression of upstream PI3K receptor genes. Pasteurized *A. muciniphila* also reduced serum inflammatory factors (Depommier et al., 2019), indicating the positive effect of dead *A. muciniphila* on systemic inflammation. Animal experiments showed that *A. muciniphila* was shown to relieve inflammation infiltration and severity by colon NLRP3 activation in DSS-induced acute colitis (Qu et al., 2021). Shi et al. (2022). demonstrated that pasteurized *A. muciniphila* lightened the gut barrier dysfunction by regulating TLR2/AMPK/NF-κB signaling. Macrophages are innate immune cells playing an essential role in the inflammatory response. Cytotoxic T lymphocytes (CTLs) are emerging as major mediators of human IBD, CTL profiling is also associated with the prognosis of IBD (Lee et al., 2011). Wang et al. demonstrated that pasteurized *A. muciniphila* (ATCC BAA-835) and it-derived amuc_1100 could alleviate colitis mouse model by decreasing colonic macrophage infiltration and CD8 + CTLs, as well as CD16/32 + macrophages in the mesenteric lymph nodes (MLN) and spleen (Wang et al., 2020a). However, the underlying molecular mechanism remains to be elucidated. Peritoneal resident macrophages have been reported to defend against microbial invasion and regulate immune responses during infections (Davies and Taylor, 2015). *A. muciniphila* supplementation could increase the abundance of peritoneal resident macrophages without affecting the abundance of neutrophils and T cells (van der Lugt et al., 2019). The data suggested that *A. muciniphila* led to activated peritoneal resident macrophages and diminished infiltrating macrophages, favoring an anti-inflammatory response rather than a pro-inflammatory effect. However, the causal mechanisms were proven only in animal models, whether they can be illustrated in humans need further study to elucidate because of the complexity of immune responses and divergences in mucin (Muc2) glycosylation of gastrointestinal tract in humans.

*Clostridioides difficile* (*C. difficile*) is a gram-positive spore-forming anaerobic bacterium and a main cause of nosocomial infections. Clostridioides difficile infection (CDI) can result in serious complications such as pseudomembranous colitis because of toxins A and B, which can promote intestinal inflammation and tissue damage (Kuehne et al., 2010). The primary treatment relies on antibiotics to eliminate active *C. difficile* bacteria, but broad-spectrum antibiotics could severely affect the healthy gut microbial balance, leading to recurrent infections and spore germination. For instance, defects in gut microbial diversity led to elevated primary bile acid (BAs) levels and decreased secondary BAs levels, which promoted *C. difficile* spore generation and prevented the growth of *C. difficile* (Kuehne et al., 2010). Therefore, restoring the gut microbiota is important for treating CDI and inhibiting recurrence (Johnson et al., 2021; Johnson et al., 2021). Both patients and mouse models with CDI showed a low proportion of *A. muciniphila* (Rodriguez et al., 2016; Deng et al., 2018; Pellissery et al., 2021). Wu et al. showed that *A. muciniphila* mitigates CDI in mice by regulating the intestinal microbiota and metabolites: it can diminish *C. difficile* abundance and toxins released in feces, reshape the gut microbiota, such as reducing the abundance of harmful *Enterococcus* and *Escherichia-Shigella* (Wu et al., 2022). It also restored the decreased secondary BAs levels in CDI mice, eased colonic inflammation by suppressing activation of TLR4/MyD88 and mTOR signaling pathways in the gut, and maintained the mucosal immune barrier (Wu et al., 2022). These effects have not yet been tested in humans and warrant further investigation.

In addition to *C. difficile*, *Salmonella*, a zoonotic pathogen, also infects the intestine of both livestock and human beings (Jantsch et al., 2011). Zhu et al. stated that *A. muciniphila* plays a protective role in *Salmonella pullorum* (*S. pullorum*) infection mice by promoting the proliferation of intestinal stem cells depending on the Wnt/β-catenin signaling pathway, thus ameliorating mucosal damage of the intestine (Zhu et al., 2020). By contrast, Ganesh et al. reported a worsening effect of *A. muciniphila* in aggravating gut inflammation in *Salmonella Typhimurium*-infected gnotobiotic mice, as evidenced by elevated histopathology scores and macrophage infiltration in cecal tissue, and increased inflammatory cytokines in colonic and cecal tissues (Ganesh et al., 2013). Therefore, *A. muciniphila* may promote *Salmonella* infection in genetically susceptible hosts. It can also be proposed that the overabundance of *A. muciniphila* reduces gut microbial diversity and results in deleterious modification of the gut environment. In addition, these two organisms simultaneously colonized in mice may disturb mucus layer homeostasis in the gut, thereby accelerating inflammatory and infectious symptoms. The molecular interactions between *A. muciniphila* and pathogens, and their consequences for host health, remain poorly understood and warrant further study.

*Acute appendicitis* is the most common surgical abdominal emergency condition. Tumors, infections, and accumulation of calcified feces in the appendix often leads to inflammation (Walter, 2021). Bacteria have been repeatedly considered the cause of inflammation. The human study conducted by Alexander et al. reported that the mean abundance of *A. muciniphila* in appendices is negatively correlated to the severity of appendicitis (Swidsinski et al., 2011). However, it’s rarely known about the causal relationship between appendicitis and microbial dysbiosis, which deserves further research.

*Cadmium (Cd)*, a toxic heavy metal, is one of the most common environmental perils in occupational areas. The gut can be severely damaged by Cd exposure, as evidenced by gut barrier dysfunction, disruption of tight junction proteins, and increased intestinal permeability, which in turn promotes inflammation and oxidative stress in the gut (Zhao et al., 2006). The abundance of *A. muciniphila* has been shown to decrease in Cd-exposed mice (Zhai et al., 2017); however, oral *A. muciniphila* could not decrease Cd absorption or accumulation in mice, as is reported by Feng et al. (2019). In addition, oral administration of *A. muciniphila* in an animal model of acute Cd exposure exhibited worse physical condition than those in acute Cd exposure group, indicating that *A. muciniphila* can accelerate mucin degradation and trigger serious inflammation in certain contexts (Feng et al., 2019).

These findings revealed that *A. muciniphila* or its components restrained intestinal inflammation, the underlying mechanisms are related to the interactions between *A. muciniphila* and cells (Figure 2). However, the protective role of *A. muciniphila* in some intestinal infections is controversial, possibly due to the disturbance of mucus layer homeostasis as a result of microbial interactions. These findings emphasized the need to further explore the specific mechanisms and molecular targets through which *A. muciniphila* modulates host immune response in the gut environment, where abundant interactions between micro-organisms exist. In addition, the causal mechanisms between intestinal diseases and *A. muciniphila* and its derivatives proven only in animal models still needs to be elucidated in human study, which is a limitation regarding their clinical therapy.

**Figure 2:**
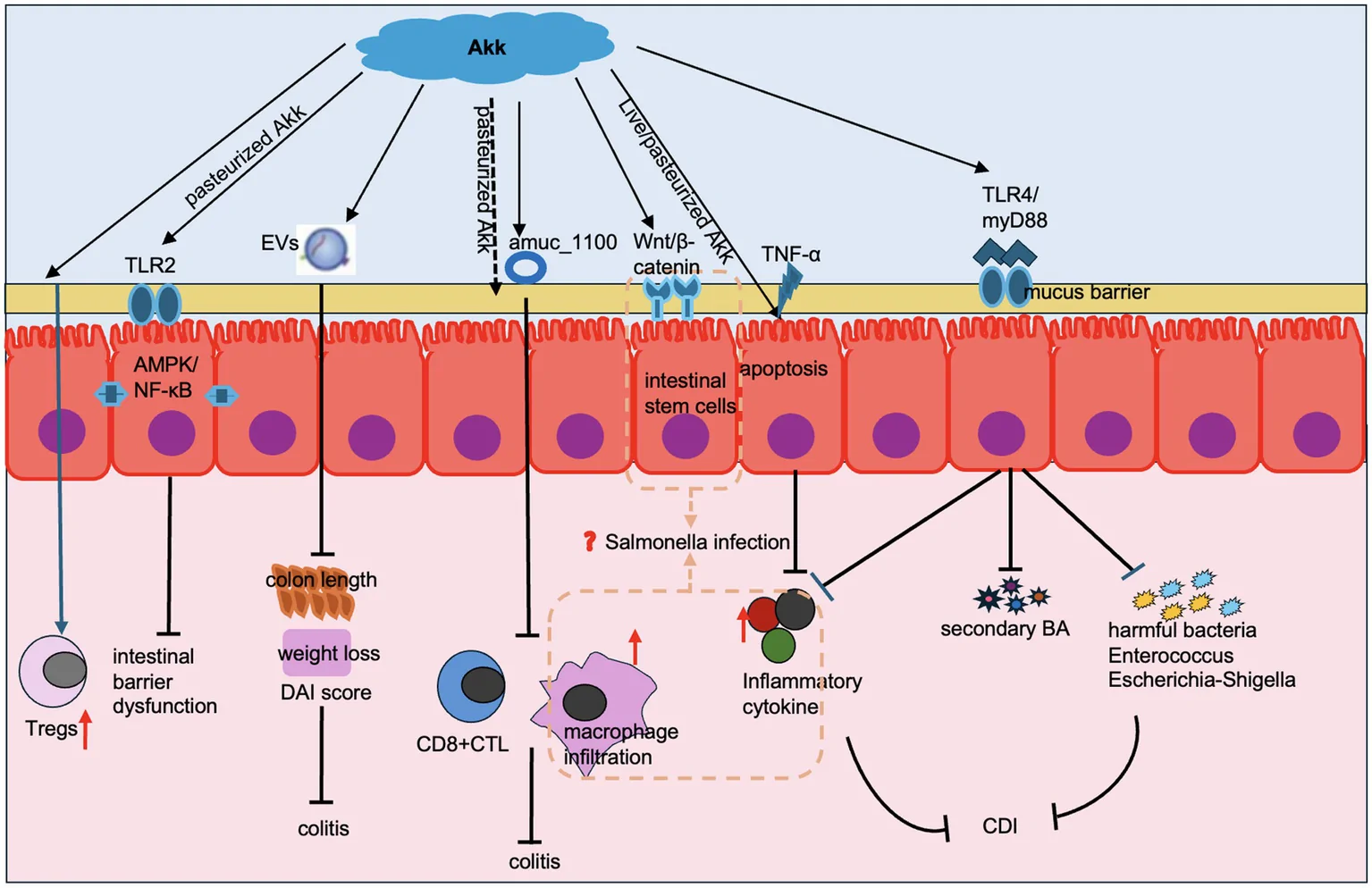
Effects of *A. muciniphila* and its derived parts on intestinal diseases.

#### *A. muciniphila* and lung, dermal diseases, atopic diseases

Improved hygiene and insufficient exposure to microorganisms in infants have been proposed as environmental contributors to allergic diseases such as asthma, atopic dermatitis (AD) and urticaria (Koga et al., 2016). Studies about the relationship between *A. muciniphila* and atopic diseases are limited. Melli et al. conducted a systematic review and stated that children with allergies have a lower abundance of *A. muciniphila*, *Clostridium*, *F. prausnitzii* and a lower bacterial diversity in the gut (Melli et al., 2016). Nabizadeh et al. showed that the frequency and relative amounts of *A. muciniphila*, *F. prausnitzii* and *Clostridium leptum* (*C. leptum*) in patients with chronic urticaria were statistically lower than those in healthy controls (Nabizadeh et al., 2017). Fieten et al. suggested that the abundance of *F. prausnitzii* and *A. muciniphila* in children with AD and a food allergy was less than that in children with AD without a food allergy. Their results implied that *A. muciniphila* may be a potential microbial biomarker associated with food allergy, which may advance in the development of future interventions (Fieten et al., 2018). Moreover, animal study reported that oral *A. muciniphila EB-AMDK19*, isolated from human feces, could mitigate dermatitis score, scratching behavior, decrease TSLP and Th2 cytokine and serum IgE levels, decline infiltrate mast cells and eosinophils in skin lesions, relieve impaired colon in 2,5-dinitrochlorobenzene (DNCB)-induced AD animal models (Lee et al., 2022). Therefore, *A. muciniphila* may be a novel therapy for AD, but large-scale isolation and industrial production of *EB-AMDK19* is a challenge and the anti-atopic function of *EB-AMDK19* in humans still needs to be confirmed. However, there were also opposing studies against the protective role of *A. muciniphila*. Human study showed that eczematous infants possess abundant *A. muciniphila* and in the animal model, higher abundance of *A. muciniphila* was associated with the susceptibility to allergic dermatitis (Zheng et al., 2016a; Sonoyama et al., 2010). They proposed that a higher abundance of *A. muciniphila* in the gut of eczematous infants might impair the integrity of the intestinal barrier, thereby raising the risk of developing eczema. Psoriasis is an immune-mediated chronic inflammatory skin disease. It was regarded as a complicated disease due to its environmental-immunity interactions based on genetics, and the pathogenesis of psoriasis is not fully understood. Tan et al. showed a reduced frequency of *A. muciniphila* in patients with psoriasis (Tan et al., 2018). Further studies are needed to clarify how the gut microbiota, including *A. muciniphila*, contributes to psoriasis and whether modulating the gut microbiota could ameliorate the disease.

Allergic asthma is a common chronic airway inflammatory disease, genetic and environmental factors alone or in combination lead to its heterogeneity (Cookson, 2004). Previous studies have reported gut microbial dysbiosis and alterations in specific microbiota in patients with asthma (Okba et al., 2018; Hevia et al., 2016). Demirci et al. reported significantly lower frequency of *A. muciniphila* and *F. prausnitzii* in children with allergic asthma than in healthy controls, and quantities of the two microbes were negatively associated with asthmatic symptoms (Demirci et al., 2019). Candela et al. showed a significant depletion in *A. muciniphila* and *F. prausnitzii* abundance in the gut of children with asthma and atopic dermatitis (Candela et al., 2012). Unfortunately, few studies have investigated the effect of oral supplementation with *A. muciniphila* on asthma in humans and animal models, and the underlying mechanisms remain largely unexplored.

Influenza infection is a common respiratory infectious disease that poses a threat to the public health. The influenza epidemic happens every year, and intermittent pandemics broke out over the past century (Cui et al., 2017). The avian-origin *influenza A* (*H7N9*) *virus* first occurred in China, substantial infectious patients and mortality have been reported (Gao et al., 2013). Previous animal studies have shown that pups or juvenile mice with *influenza A virus* infection showed increased abundance of *A. muciniphila* in the gut (Fuglsang et al., 2018). A study by Zhang et al. reported a similar trend in mouse model of *H7N9 virus* infection, suggesting an adverse effect of *A. muciniphila* (Zhang et al., 2020a). Interestingly, their colleagues demonstrated that oral administration of *A. muciniphila* suppressed *H7N9 virus* proliferation *in vivo*, ameliorated pathologic changes in the lung and clinical symptoms, and prevented cytokine release in the lungs in mice with *H7N9* infection (Hu et al., 2020). They further investigated that orally pasteurized *A. muciniphila* declined mortality and weight loss in mice infected with the *PR8 influenza virus* (Hu et al., 2020), suggesting a broad-spectrum anti-influenza effect of *A. muciniphila*. These data suggest that the benefits of *A. muciniphila* may depend on specific components rather than on its proliferation in the gut. Whether *A. muciniphila* plays a beneficial role in humans infected with influenza remain to be fully understood, and this uncertainty currently limits its clinical translations in *influenza A virus* infection.

These results implied the potential therapeutic role of *A. muciniphila* in lung, dermal, and atopic diseases. However, there were no studies elucidating the underlying mechanisms in these settings, which deserves further research for the use of clinical therapy.

#### *A. muciniphila* and rheumatic diseases

Regulation of the immune system by microbes, especially intestinal microbiota, is increasingly regarded as a vital factor in the onset, course, and outcome of rheumatic diseases. The interactions among microbiota, genetic predisposition and environmental factors, are considered as an important trigger for rheumatic diseases (Ostrov and Amsterdam, 2017). Accumulating evidence suggested that the gut microbiota plays a role in activation or induction of Treg and Th17 cells, both of which might affect the development of autoimmune disease (Scofield, 2014). Previous study reported the associations between *Porphyromonas gingivalis* (*P gingivalis*), a specific member of the oral microbiota, and the pathophysiological processes of rheumatoid arthritis (Rosenstein et al., 2004). The connection between the microbiota and other rheumatic autoimmune diseases remains to be established. Recent studies shed light upon the potential correlations between intestinal microbial dysbiosis and different types of arthritis, including juvenile idiopathic arthritis, juvenile spondyloarthritis, psoriatic arthritis and rheumatoid arthritis (Stoll et al., 2014; Tejesvi et al., 2016; Maeda et al., 2016; Scher et al., 2015). Specifically, children with juvenile spondyloarthritis showed significantly elevated abundance of fecal *A. muciniphila*, and mice with an abundance of *A. muciniphila* ≥ 2% exhibited more severe ankle thickness than mice with abundance < 2% in enthesitis-related arthritis (Stoll et al., 2014). In a rat model of Spondyloarthritis, increasing colonization of gut *A. muciniphila* and a higher proportion of IgA-coated fecal bacteria were statistically associated with HLA-B27 expression and arthritis development (Asquith et al., 2016). Stoll et al. demonstrated that the administration of *A. muciniphila* to Altered Schaedler’s flora (ASF) led to a small but statistical elevation in ankle swelling in comparison with mice that received ASF alone in arthritis model (Stoll et al., 2019). By contrast, Scher et al. reported that adults with psoriatic arthritis had a decreased gut abundance of *A. muciniphila* compared with healthy controls (Scher et al., 2015). These data implied that the relationship between microbiota and autoimmune rheumatic diseases is an area of high interest. Thus, the precise role of *A. muciniphila* in arthritis still needs to be elucidated before it can be considered for clinical application.

#### *A. muciniphila* and liver diseases

The gut microbiota-liver signaling axis contributes to liver diseases, such as alcoholic liver disease (ALD), nonalcoholic fatty liver disease (NAFLD), and hepatocellular carcinoma (HCC) (Vanni and Bugianesi, 2009; Ezzaidi et al., 2019). *A. muciniphila* is an important player in various hepatic diseases owing to its ability to promote cholesterol transportation, restore abnormal bile acid metabolism, and regulate the pathways of inflammatory signaling pathways in the gut-liver axis (Rao et al., 2021) (Keshavarz Azizi Keshavarz Azizi Raftar et al., 2021a). In ALD, ethanol exposure led to the defect in intestinal *A. muciniphila* abundance in both humans and mice, and oral *A. muciniphila* could alleviate experimental ALD mice by reducing hepatic injury, steatosis, and infiltration of neutrophils; enhancing mucus thickness and the expression of tight-junction protein (Grander et al., 2018). Grander et al. also found that live *A. muciniphila* could relieve liver damage and restore intestinal barrier integrity in animal models of alcohol-induced liver injury (Grander et al., 2018).

##### Animal and *in vitro* studies

Metabolic dysfunction-associated fatty liver disease (MAFLD), previously called nonalcoholic fatty liver disease (NAFLD), is regarded as a wide range of liver diseases, including nonalcoholic steatohepatitis (NASH), simple steatosis (NAFL), cirrhosis, fibrosis, and HCC. Shi et al. found changes in the gut microbiota in saccharin/sucralose-induced NAFLD animal models. For instance, they found a reduced abundance of *A. muciniphila* in the cecal contents of mice, resulting in increased intestinal permeability and elevated serum lipopolysaccharide level, potentially driving systemic inflammation and NAFLD development in mice (Shi et al., 2021). Another study showed that *A. muciniphila* facilitated the transportation of l-aspartate from the gut to the liver, stimulating LKB1-AMPK signaling and lipid oxidation to relieve MAFLD in mice (Rao et al., 2021). As fatty liver disease is the main cause of hepatitis, *A. muciniphila* could prohibit fatty liver disease in obese mice by maintaining gut homeostasis and modulating triglyceride (TG) synthesis in the liver(Kim et al., 2020). Various liver disorders can progress to liver fibrosis through excessive accumulation of fibrillar extracellular matrix (ECM) in the liver, HSC activation and facilitated hepatocellular dysfunction (Parola and Pinzani, 2019). *In vitro*, heat-killed *A. muciniphila* exhibited anti-fibrotic effects on HSCs, such as increasing PPARγ expression, reducing the expression of TGF-β, TLR4 and Col1 in LPS-incubated LX-2 cells (Keshavarz Azizi Keshavarz Azizi Raftar et al., 2021a). Insulin-like growth factor (IGF) is an anti-inflammatory factor, which triggers the development of chronic liver disease (CLD) and primary liver cancer. A previous animal experiment reported that oral administration of live and pasteurized *A. muciniphila* and its EVs significantly restored IGF gene expression (Raftar et al., 2022). However, these findings were not validated in human study, which is a prerequisite for the clinical translation of *A. muciniphila*.

Viral infection, alcohol consumption, high-fat diet, and hepatitis can all cause liver damage (Wu et al., 2017). Liver inflammation is one of the main causes of hepatic tissue damage and liver disease, triggering the progression of liver injury, liver fibrosis and eventually HCC (Wang et al., 2020b). Inflammation can be promoted by different pathogen-associated molecular patterns (PAMPs) and damage-associated molecular patterns (DAMPs) via toll-like receptors (TLRs) in the liver (Krenkel and Tacke, 2017). *In vitro*, heat-killed *A. muciniphila* could reduce TLR-2 and TLR-4 gene expressions in LPS-incubated LX-2 cell lines and cause HSC regression by elevating PPAR-γ mRNA levels, thus exerting an anti-fibrotic role in restraining liver fibrosis (Keshavarz Azizi Keshavarz Azizi Raftar et al., 2021a). Another study reported a protective role of pasteurized and live *A. muciniphila* and its EVs in HFD/CCl4-induced liver injury models, as demonstrated by reduced fibrosis markers (e.g., TGF-β, PDGF, α-SMA, TLR-2 and TIMP), ameliorated inflammation, and restored gut microbiome (Keshavarz Azizi Keshavarz Azizi Raftar et al., 2021b). Similarly, Shahrbanoo et al. reported the anti-inflammatory effect of *A. muciniphila* and it-derived EVs in mouse model of HFD/CCL4-induced liver injury, they can decreased IL-6 and IL-1beta gene expressions, increased IL-10 expression, decreased TLR-9 and TLR-5 expressions in liver tissue. Their previous study showed that *A. muciniphila* and its EVs increased the expression of PPAR-α and PPAR-γ in the adipose tissue of HFD-fed mice, improving fatty acid oxidation and energy metabolism (Ashrafian et al., 2019). Owing to the inhibitory role of PPAR activation in fatty acid accumulation and the regulation of inflammatory responses, these authors proposed that *A. muciniphila* and its EVs possesses excellent protective effects against liver injury by restoring the intestinal barrier integrity and reducing mRNA expressions of TLR-9 and TLR-5, thereby resulting in PPAR-α and PPAR-γ activation in the liver (Raftar et al., 2022). Concanavalin A (Con A) injection is widely used to establish a liver injury model resembling autoimmune liver diseases and viral hepatitis, the features of which include T cell-mediated hepatocellular death and inflammatory infiltration (Heymann et al., 2015). Wu et al. reported that administration of live *A. muciniphila* for 14 days reduced neutrophils and macrophages infiltration in an immune-mediated liver injury mouse model (Wu et al., 2017). Wu et al. suggested that *A. muciniphila* exhibited a protective role in mice with immune-mediated liver injury by prohibiting inflammation and hepatocellular death (Wu et al., 2017). Animal experiments further showed that *A. muciniphila* could significantly ameliorate acetaminophen (APAP)-induced hepatocellular apoptosis by restraining MAPK activation, maintaining GSH/GSSG equilibrium, and inhibiting inflammation, oxidative stress, and neutrophil and macrophage infiltration in the liver. Moreover, the beneficial effect of *A. muciniphila* was accompanied by PI3K/Akt signaling activation and mediated by the regulation of intestinal microbial community and microbial metabolic function (Xia et al., 2022). The effect of *A. muciniphila* and it-derived EVs on liver inflammation, however, has not yet been investigated in humans.

These studies provide new insights into the pathogenesis of live diseases as a result of gut microbial dysbiosis and stress the significant role of *A. muciniphila* and its derivatives in the treating and preventing live diseases (Figure 3). However, the clinical translation of *A. muciniphila* and it-derived EVs was limited by the failure to identify the contents of *A. muciniphila*-derived EVs to illustrate the exact beneficial mechanism of these bacterial derivatives in the liver diseases and the failure to confirm their effects on liver diseases in humans, as well as their potential risks and adverse effects.

**Figure 3:**
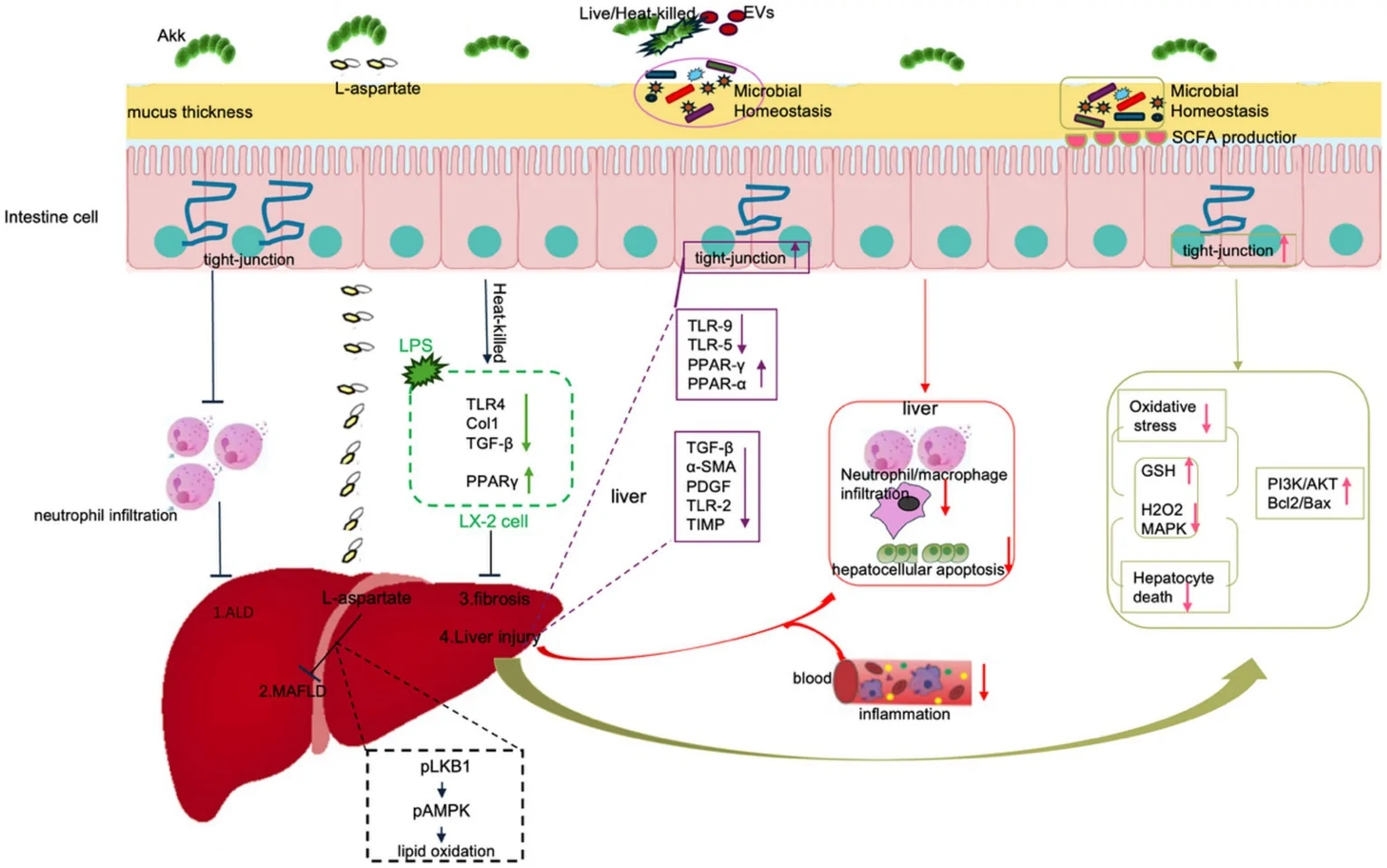
Effects of *A. muciniphila* and its derived parts on liver diseases.

##### *A. muciniphila* and neurological disorders

The relationship between brain and the intestinal tract, termed the gut-brain axis, has been a hot point of numerous studies for decades (Dinan and Cryan, 2017). The bidirectional communications between the two organs are extremely complex and makes up of the enteric nervous system, including neuroendocrine and neuroimmune signaling pathways, the sympathetic and parasympathetic autonomic nervous system branches (Foster et al., 2017; Cryan et al., 2019). Growing evidence has shown that the gut microbiome plays an important role in the pathogenesis of neurological and neuropsychiatric disorders by influencing neuronal transmission, myelination, synaptic plasticity, and complex host behaviours (Sampson et al., 2016; Sharon et al., 2019; Blacher et al., 2017; Sharon et al., 2016).

Neurological disorders are common among veterans who have experienced the Gulf War. Those with Gulf War illness (GWI) experience persistent symptoms, including neurological disorders, headaches, muscle weakness, and memory loss. It has been demonstrated that the low proportion of *A. muciniphila* is significantly correlated with GWI symptom persistence via decreased Brain-Derived Neurotrophic Factor (BNDF) levels and NLRP3-mediated neuroinflammation (Kimono et al., 2020). Gut microbial dysbiosis was also associated with autism spectrum disorder (ASD), a complex neurodevelopmental disorder with low proportions of *A. muciniphila* and *Bifidobacterium* spp. in the feces of patients (Wang et al., 2011). Major depressive disorder (MDD), which has recently been linked to the gut-brain axis, is characterized by diminished interest, depressed mood, cognitive impairment, and vegetative symptoms such as reduced appetite and disturbed sleep (Otte et al., 2016). Globally, it has the second-largest disease burden. Increasing studies have reported a specific link between depression and gut microbial dysbiosis and metabolic abnormality (Jiang et al., 2015; Fung et al., 2017). Germ-free mice colonized with “depressive” fecal microbiota from MDD patients led to depressive-like behaviors, compared with those colonized with “healthy” microbiota from healthy controls (Zheng et al., 2016b). Stress is a major risk factor of depression. In a modified mouse model of chronic social defeat stress, McGaughey et al. found the relative abundance of genus *Akkermansia* was significantly lower than that in control mice (McGaughey et al., 2019). However, they did not quantify the species *A. muciniphila* in the mouse model. Ding et al. reported an antidepressant effect of *A. muciniphila* in chronic restraint stress (CRS) induced mouse models, *A. muciniphila* could modulate gut microbiota and metabolites, affect hormone levels, and promote the synaptic signaling pathway and neuronal connections (Ding et al., 2021). Chronic unpredictable mild stress (CUMS) is a common model for depression in rodents. Animal experiment suggested that an outer protein of *A. muciniphila*, Amuc_1100, could reduce 5-HT levels in the serum and colon, restore the gut microbiota dysregulation, and rescue the reduced BDNF in the hippocampus, thereby ameliorating CUMS-induced depression-like behavior (Cheng et al., 2021). *A. muciniphila* also exerts multiple functions in neurological processes. For instance, one of the *A. muciniphila* subtypes was demonstrated to protect against neurodegenerative process in a mouse model of high-fat diet-induced obesity and diabetes through improving spatial memory, elevating Nissl bodies in neurons, and restoring tryptophan metabolism (Wu et al., 2020). Yang et al. reported an unexpected pivotal effect of *A. muciniphila* on restoring hippocampal-dependent cognitive function, and reversing hippocampal neuronal development, microgliosis, long-term potentiation (LTP), and inflammatory cytokines expressions in the hippocampus of HFD-fed mice (Yang et al., 2019).

The gut-brain axis is regarded as a possible cause of Alzheimer’s disease (AD) due to the shift of gut microbial community toward an inflammation-related profile, which was possibly related to disease progression and severity (Bäuerl et al., 2018). Gut microbial alterations were detected before the onset of AD and reported in patients with mild cognitive impairment (MCI). In a human study, the abundance of genus Akkermansia in the gut was statistically higher in the AD group and was positively correlated with medial temporal atrophy (MTA) (Li et al., 2019a). It’s worth to note that the genus Akkermansia contains various species, such as *A. muciniphila, Akkermansia biwaensis, and Akkermansia glycaniphila,* the human study specified the links of the abundance of genus *Akkermansia* to MTA in AD, whether *A. muciniphila* specifically caused MTA and the underlying mechanisms warrant further research to elucidate. In animal models of AD, *A. muciniphila* improved cognitive dysfunction, amyloid pathology, and anxiety-related behaviors (Ou et al., 2020). The results of human and animal studies at the present did not give a clear picture of the contribution of *A. muciniphila* to AD aetiology. Parkinson’s disease (PD) is a neurodegenerative disorder characterized by motor symptoms, including muscle rigidity, slowness, tremors, and difficulty in controlling movement. Its pathological hallmarks include loss of dopaminergic neurons in the substantia nigra (SN) pars compacta (SNpc), aggregation of misfolded α-synuclein within Lewy bodies (LBs) and causative monogenic mutations (Bekris et al., 2010; Spillantini et al., 1997). The molecular and cellular mechanisms of PD have attracted much attention, especially triggered by the discovery of the associations between enteric nerves and enteroendocrine cells through α-synuclein expression. Enteroendocrine cells, which have neuron-like functions, are part of the intestinal epithelium and are exposed to the gut microbiota, supporting the notion that PD pathology may arise in the gut and spread to the brain along cell-nerve connections (Chandra et al., 2017). Patients with PD showed gut microbial dysbiosis, and the abundance of *A. muciniphila*, one of the mucin-degrading *Verrucomicrobiaceae*, are significantly increased (Hill-Burns et al., 2017; Keshavarzian et al., 2015; Li et al., 2017; Scheperjans et al., 2015). Oral *A. muciniphila* induces α-synuclein aggregation and mitochondrial calcium overload in an enteroendocrine cell line, providing experimental support for a gut microbial dysbiosis in PD (Amorim Neto et al., 2022). However, it remains to be addressed whether α-synuclein aggregation of enteroendocrine cells in the mouse gut caused by *A. muciniphila* could be transferred to enteric neurons and, thereby, promote the α-synuclein pathology progression in PD. Aging is an important risk factor for many neurodegenerative diseases such as PD and AD. Ten percent of the elderly (aged ≥65 years) are diagnosed with AD, and its prevalence rises with age. Unfortunately, efficient therapies for age-related neurodegenerative diseases are limited, causing an increasing socioeconomic burden. Some studies have investigated the gut microbial community of long-lived humans (Biagi et al., 2016; O’Toole and Jeffery, 2015). Clea et al. found that progeroid mice exhibited a loss of *A. muciniphila* in the gut, and oral *A. muciniphila* significantly increased the lifespan of progeroid mice (Bárcena et al., 2019), thereby broadening our understanding of the pro-health effects of *A. muciniphila* in aged mice (Bodogai et al., 2018). Therefore, we may hypothesize that individuals with extreme longevity possess more beneficial intestinal microbiota. Amyotrophic lateral sclerosis (ALS) is a neurodegenerative disease characterized by the premature death of motor neurons and the patients with ALS has only three-five years of lifespan from diagnosis. *A. muciniphila* was demonstrated to improve motor function, reduce brain atrophy, and prolong the lifespan of ALS animal models (Blacher et al., 2019).

Multiple sclerosis (MS) is an autoimmune disease of the central nervous system (CNS), involving demyelination, oligodendrocyte loss, reactive gliosis, and axonal degeneration (Stadelmann et al., 2011; Ransohoff et al., 2015). Previous study reported an altered gut microbiota in MS patients, and *A. muciniphila* could ameliorate the experimental autoimmune encephalomyelitis (EAE) mouse model of MS by inducing Tregs depending on increased TGF-β, reduced IL-6 and IL-1β release by DCs (Liu et al., 2019).

Figure 4 summarizes the mechanisms through which *A. muciniphila* acts in neurological diseases. However, these human data are still preliminary and observational, and the experimental evidence are not sufficient to make a treatment recommendation for the neurological disease. Future studies are needed to elucidate the suggested microbial associations and investigate the causal effects of potential microbial modulators in the nervous system.

**Figure 4:**
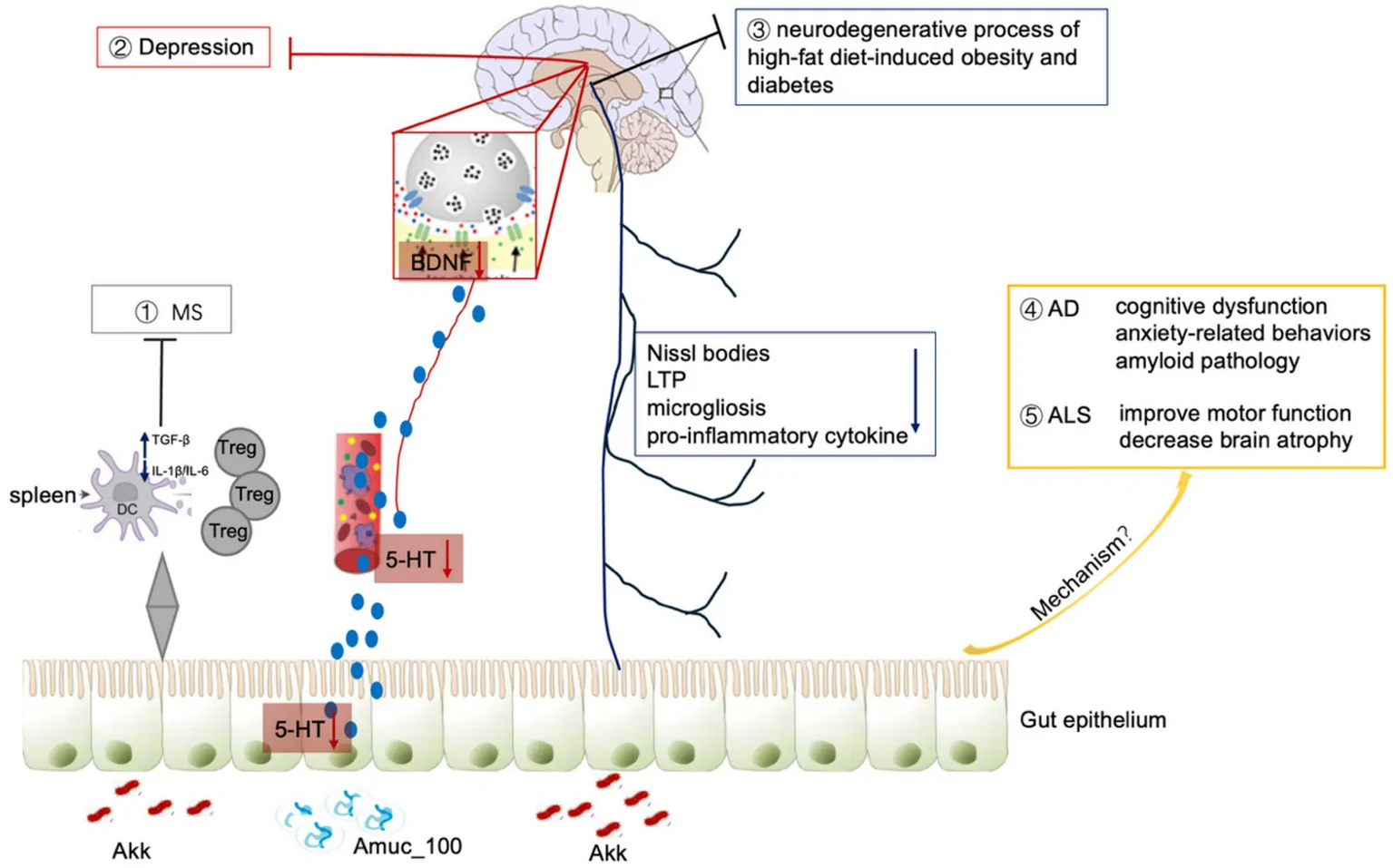
Effects of *A. muciniphila* and its derived parts on neurological diseases.

#### *A. muciniphila* and skeletal disorders

There is now broad consensus that the gut microbiota exerts a profound effect on health (Gilbert et al., 2018). It regulates bone homeostasis, although the underlying mechanisms are still being defined. A previous study reported that the administration of a low dose of antibiotics to mice at weaning resulted in a shift in gut microbial composition and high bone mass (Cho et al., 2012). The potential mechanisms underlying the effect of the gut microbiota on bone metabolism include altered production of microbial metabolites, changes in nutrient absorption, modulation of immune responses, and effects on endocrine and hormonal secretion (Zheng et al., 2025; Lepage et al., 2013; Rastelli et al., 2019). Probiotic treatment can inhibit sex steroid depletion-induced bone loss in mice by maintaining the gut barrier and restraining inflammation and osteoclastogenesis (Britton et al., 2014; Li et al., 2016b). In an animal study, Liu et al. demonstrated that *A. muciniphila* could inhibit the inflammatory response at the early stage of fracture site and promote type H vessel formation in angiogenesis and callus, thus facilitating in fracture healing and bone formation (Liu et al., 2020), suggesting that *A. muciniphila* is a promising therapeutic strategy for bone healing.

Osteoporosis, an age-related bone metabolic disease, is a common bone-related disease in the elderly, especially in postmenopausal women. It has multiple causes, and the gut microbiota, which is closely linked to bone homeostasis, contributes to its development and progression (Zheng et al., 2016b). The gut microbiota may be a candidate treatment for osteoporosis. An animal study showed that the direct replenishment of *A. muciniphila* could restore the ovariectomy (OVX)-induced abnormal bone metabolism and protect against osteoporosis. They further investigated the underlying mechanism and showed that the bone-protective effects of *A. muciniphila* relied on the release of EVs. Animal tracking experiments showed that the *A. muciniphila*-derived EVs can accumulate in bone tissues and relieve the OVX-induced osteoporotic phenotypes by inhibiting osteoclast formation, elevating osteogenic activity, enhancing bone mass and strength (Liu et al., 2021a). The evidence suggested that *A. muciniphila* can contact with the host bone cells through the release of EVs that cross the intestinal barrier into the systemic circulation and reach the bone tissues to regulate osteoblastic bone formation and osteoclastic bone resorption. Moreover, the EVs contain many molecules that exert functions on bone health and the components performing the protective role still require future research.

In summary, restoring gut microbial homeostasis is predicted to improve bone health. Further study of the mechanism underlying the impact of gut microbiota on bone health and deeper understanding of the gut-bone axis are important for targeting bone-related diseases with a balanced gut microbiota. It’s also worth to note that the protective role of *A. muciniphila* and it-derived EVs on abnormal bone metabolism was proven only in animal models, it’s still unknown about their effect on bone-related diseases in humans, which limits their clinical application and needs future research.

#### *A. muciniphila* and oral diseases

Periodontitis is a chronic inflammatory disease with more than 700 million cases globally (GBD 2017 Oral Disorders Collaborators et al., 2020). This disease is featured by inflammation, which causes damage of the tooth-supporting apparatus, including alveolar bone and cement, finally resulting in tooth loss (Kinane et al., 2017). Periodontitis is caused by multi-layer homeostatic failure including dysbiosis of microbiota, host immune response, periodontal ligament stem cells and bone resorption (Zhou et al., 2026). *P gingivalis* is strongly associated with periodontal inflammation, destruction of gingival tissue, and alveolar bone loss through interactions with gingival epithelial cells, triggering the accumulation of neutrophils and M1-like macrophages in the gingival tissue (Bryzek et al., 2019; Huck et al., 2017; Huck et al., 2018; Sochalska and Potempa, 2017). In patients with severe periodontitis, the abundance of *A. muciniphila* is totally absent in the oral cavities (Coretti et al., 2017). Administration of *A. muciniphila* significantly reduced inflammation of periodontal tissue and destruction of calvarial bone in mice, upregulated the expression of cell–cell adhesion and tight junction markers, and promoted IL-8 expression in gingival epithelial cells, ultimately ameliorating bone loss caused by *P gingivalis* inoculation (Huck et al., 2020). Mulhall et al. showed that *A. muciniphila* or its outer membrane protein Amuc_1100 could induce beneficial M2 macrophages in submandibular lymph nodes and elevate IL-10 expressions in gingival tissues and macrophages, thus relieving *P gingivalis*-induced alveolar bone loss in experimental periodontitis mice. In their study, the gingival tissue in *A. muciniphila* and Amuc_1100-treated mice exhibited elevated expression of the T-cell chemoattractant CXCL10, which was also associated with the decreased alveolar bone loss detected in these groups (Mulhall et al., 2020). We may propose that *A. muciniphila* and Amuc_1100 could promote bacterial clearance in gingivalis infection context through the recruiting Th1 cells to the gingival tissue depending on T-cell chemoattractant CXCL10. Therefore, *A. muciniphila* and Amuc_1100 may have the potential to be promising therapies for preventing and resolving periodontitis. Further research is required to elucidate the underlying cellular mechanisms. The ability of *A. muciniphila* to regulate host responses through both epithelial cell and immune standpoints could pave the way for its function as an adjunctive therapy to non-surgical periodontal treatment. Undeniably, the complex immune context in humans cannot be completely replicated by animals and *in vitro* environment, which limits the clinical translation of *A. muciniphila* and Amuc_1100.

#### *A. muciniphila* and cardiovascular diseases

Atherosclerosis is a chronic inflammatory disease in which inflammation and hypercholesterolemia are important etiological factors (Weber and Noels, 2011). Bacterial infection has been regarded as one of the contributors of inflammation of atherosclerosis (Davidson et al., 1998; Muhlestein et al., 1998). Indeed, bacterial DNA has been detected in atherosclerotic plaques, and these observed bacterial taxa were also detected in the gut of the same person (Koren et al., 2011; Ott et al., 2006). These studies prompted us to speculate whether the gut is the source of the microbiota in the plaques. The microbiota at these sites may be one of the underlying mechanisms of bacterial infection in the plaque, which likely affect plaque stability as well as the development of cardiovascular disease.

A recent mouse study showed that oral *A. muciniphila* reversed western diet-induced inflammation in local atherosclerotic lesions and circulation, as demonstrated by reduced macrophage infiltration, release of chemokines and pro-inflammatory cytokines (Li et al., 2016a). However, *A. muciniphila* had no effect on hypercholesterolemia and other metabolic profiles in mice, implying that *A. muciniphila* may function as an anti-atherosclerotic agent primarily through its anti-inflammatory function. The underlying molecular mechanisms remain unclear, and the components of *A. muciniphila* that may influence lipid metabolism in atherosclerosis have yet to be identified.

The animal study uncovered a key link between *A. muciniphila* and cardiovascular diseases, the mechanisms established in animal models are still a long way from clinical application.

#### *A. muciniphila* and metabolic diseases

### Type 2 diabetes (T2D)

#### Human studies

In recent years, the associations between *A. muciniphila* and metabolic diseases has yielded substantial positive results. The relative abundance of *A. muciniphila* in the gut is significantly reduced in obese humans during prediabetes and diabetes (Le Chatelier et al., 2013; Ellekilde et al., 2014; Karlsson et al., 2012; Allin et al., 2018), whereas caloric restriction increases the abundance of *A. muciniphila*, which is accompanied by an improvement of cardiometabolic parameters (Dao et al., 2016). However, the opposite relationship was observed in lean adults with T2D, who had an enriched *A. muciniphila* abundance in the gut compared with non-diabetic controls (Qin et al., 2012). A study of normal-weight 70-year-old European women likewise argued against a role for *A. muciniphila* in classifying women with T2D (Karlsson et al., 2013). Therefore, the role of *A. muciniphila* in obese humans is still ambiguous, and we may also speculate a potential protective role of *A. muciniphila* in T2D patients and obese people.

#### Animal and *in vitro* studies

Growing evidence showed that low-grade inflammation, oxidative stress, gluco/lipotoxicity, and endoplasmic reticulum (ER) stress cause the defect of β-cells and insulin resistance, thereby playing an essential role in T2DM pathogenesis (Eizirik et al., 2013). The contribution of destructive gut permeability in T2DM has attracted increasing attention (Creely et al., 2007; Ding et al., 2010). Globally, live and pasteurized *A. muciniphila* and it-derived EVs can decrease fat mass, hyperglycemia, dyslipidemia, glucose intolerance, and insulin resistance (IR) in obese and diabetic mice. Compared to the bacterium itself, its EVs showed more significant anti-inflammatory effects on the epididymal adipose tissue (EAT) of obese mice, as evidenced by reduced TLR-4 expression and inflammatory cytokines (TNF-α, IL-6) in EAT (Ashrafian et al., 2019). In addition, pasteurized *A. muciniphila* enhanced the above capacity of the bacterium by modulating the host metabolomic profile of urine and energy absorption of the gut. Amuc_1100 is a specific outer membrane protein of *A. muciniphila*, it remains stable at temperatures used for pasteurization, partly mediates the beneficial effects of the *A. muciniphila* and exhibits interaction with TLR 2. Amuc_1100 was demonstrated to induce a lower body weight and fat mass gain than that in HFD-fed mice, it could also improve hypercholesterolemia, glucose tolerance, insulin-induced phosphorylation of the insulin receptor (IR) and endotoxemia in HFD-fed mice. Moreover, Amuc_1100 reduces gut permeability in HFD-fed mice, as evidenced by increased expressions of CLDN3 (encoding claudin 3), OCLN (encoding occludin) in the jejunum (Plovier et al., 2017). Recently, Yang et al. isolated three new *A. muciniphila* strains (EB-AMDK-10, 19, 27) from the human gut and showed that these strains decreased fat mass, body weight gain, calorie intake, serum total cholesterol (TC) levels, adipogenesis/lipogenesis, and inflammatory insults. Nonetheless, these pasteurized *A. muciniphila* strains restored the impaired gut integrity and improved hepatic functions, glucose homeostasis, and insulin resistance in HFD-induced obese mice (Yang et al., 2020). Therefore, the live and pasteurized *A. muciniphila*, together with derivatives such as Amuc_1100, thus represent a step toward the development of *A. muciniphila*-based medicines that could target T2D and other diseases involving impaired intestinal barrier function.

T2D is characterized by a chronic low-grade inflammation that contributes to insulin resistance. A high-fat diet (HFD) could alter gut microbial diversity and intestinal wall permeability, prompting LPS to pass through the impaired gut barrier to enter the systemic circulation and causing metabolic endotoxemia. Excessive LPS can induce ER stress in insulin-responsive cells (Liang et al., 2013). Zhang et al. reported that oral live *A. muciniphila* could not protect against systemic inflammation in normal rats; but, live and pasteurized *A. muciniphila* significantly improve systemic inflammation and reduced inflammatory cytokines (LPS, TNF-α) in diabetic rats. The reduced LPS levels in diabetic rats also implied the function of *A. muciniphila* in decreasing gut permeability (Zhang et al., 2018). LPS binding protein (LBP) is an acute-reaction glycoprotein binding LPS with high affinity and transport LPS to the TLR4 receptor complex, subsequently activating JNK activation, NF-κB signaling pathway, and inducing pro-inflammatory cytokines production (Pfalzgraff and Weindl, 2019; Zweigner et al., 2006; Tsaousidou et al., 2014). Serum LBP is a marker of inflammation and is closely associated with insulin resistance. JNK is known to combine with IRS1 and activate Ser307 phosphorylation, causing inactivation of AKT and insulin signaling (Hirosumi et al., 2002). In addition, IKK-NF-κB pathway activation exerts an essential role in insulin resistance and metabolic abnormalities (Hirosumi et al., 2002). It has been demonstrated that *A. muciniphila* administration significantly reduced LBP levels in plasma of chow diet-fed mice, suppressed JNK phosphorylation, and elevated NF-KB inhibitor protein and IKBA protein levels in muscle and liver (Zhang et al., 2018), thereby alleviating metabolic endotoxemia, triggering insulin signaling activation the liver and muscle, and ameliorating global glucose tolerance. The intestinal endocannabinoid system has also been shown to control the gut barrier and inflammation (Muccioli et al., 2010; Cani, 2012). Oral *A. muciniphila* significantly elevated the production of intestinal endogenous 2-arachidonoylglycerol (2-AG), 2-oleoylglycerol (2-OG), and 2-palmitoylglycerol (2-PG) in obese mice (Everard et al., 2013). Of these, 2-AG and 2-PG can restrain metabolic endotoxemia and exert an anti-inflammatory effect on systemic inflammation (Alhouayek et al., 2011). 2-OG promotes intestinal L-cells to release gut peptides from glucagon-like peptide-1(GLP-1) and GLP-2 (Hansen et al., 2011), which could lower blood glucose and inhibit β-cell apoptosis. Therefore, we may hypothesize that modulation of the endocannabinoid system also underlies the anti-inflammatory effects induced by *A. muciniphila* in T2D and obesity.

Increased gut permeability is closely associated with T2D and promotes the penetration of bacteria across the gut barrier, causing metabolic endotoxemia and low-grade systemic inflammation (Fan and Pedersen, 2021). Impaired intestinal mucosal permeability also occurs in obesity, *A. muciniphila* and its EVs could restore the increased gut barrier permeability in HFD-induced obese mice by elevating tight junction protein expression (ZO-1, CLDN-1, OCLDN) and reducing CLDN-2 expression in the colon, moreover EVs had a better effect (Ashrafian et al., 2019). Chelakkot et al. demonstrated that *A. muciniphila*-derived EVs could reduce food intake and body weight gain, improve glucose tolerance, decrease adipose inflammatory genes expression (TNF-α and IL-6), diminish gut permeability through regulation of tight junction expression in HFD-induced diabetic mice (Chelakkot et al., 2018). Moreover, treatment of the outer membrane protein of *A. muciniphila* (Amuc_1100) and three new pasteurized *A. muciniphila* strains resulted in lower body weight and fat mass gain, lower plasma LDL cholesterol, improved glucose tolerance, increased genes associated with the intestinal barrier, when compared to that in untreated HFD-fed mice (Plovier et al., 2017; Yang et al., 2020).

Elevated free fatty acids (FFA) in plasma adversely impair the secretion and function of insulin in pancreatic β-cells (lipotoxicity) and contribute to the progression from normal glucose to overt hyperglycemia. In return, hyperglycemia aggravates the above process, leading to a vicious cycle for the diabetic state known as gluco-lipotoxicity (Del Prato, 2009; Véret et al., 2014). In streptozotocin (STZ) -induced diabetic rats, oral administration of *A. muciniphila* reduced hepatic glycogen levels and increased HDL-C levels, intensifying resistance to gluco/lipotoxicity (Zhang et al., 2018). Zhao et al. reported that *A. muciniphila* supplementation significantly declined the gene expression of sterol regulatory element-binding proteins (SREBP1c) and fatty acid translocase (CD36) which were involved in lipogenesis and the transition of fatty acids both in the liver and muscle, and also improved fat deposition in hepatic tissue and muscle. Their study suggested that *A. muciniphila* may reduce lipid accumulation in the hepatic tissue and muscle, thereby improving insulin sensitivity (Zhao et al., 2017).

Insulin is formed in the endoplasmic reticulum (ER) and initially translated into proinsulin. ER homeostasis is essential for maintaining β-cell functions and metabolic homeostasis. ER stress contributes to insulin resistance and the activation of inflammatory pathways in innate immune system (Hotamisligil, 2010), and it is closely linked to obesity and T2D (Lee and Lee, 2022; Lee and Ozcan, 2014). ER membranes contain key lipogenic enzymes involved in lipid biosynthesis, which further damages insulin signaling (Lee et al., 2008). Zhao et al. showed that oral administration of *A. muciniphila* could reduce ER stress proteins, such as immunoglobulin heavy chain-binding protein/glucose-regulated protein 78 (BiP/GRP78) and PERK in the liver and ER stress transducers genes (CHOP, TRIB3) in the colon(Zhao et al., 2017). Oxidative stress and free radical generation also play vital roles in diabetes mellitus because of the damage to β-cell function, lipid metabolism, and insulin secretion(Darenskaya et al., 2021; Ighodaro, 2018). Under oxidative stress conditions, a late-stage lipid oxidation byproduct, MDA, was significantly elevated in plasma, the renal cortex, mesangial cells, and proximal tubule cells (Cheng et al., 2013). In diabetic rats, live and pasteurized *A. muciniphila* could reduce surplus MDA, indicating the protective role of *A. muciniphila* against oxidative damage in diabetes (Zhang et al., 2018). Hypofibrinolysis is a key abnormality in diabetes that contributes to a hypercoagulated state and the adverse vascular outcomes in diabetes patients. Plasminogen activator inhibitor-1 (PAI-1), a key regulator of fibrinolysis, was significantly reduced by *A. muciniphila* in STZ-diabetic rats, accompanied by a decline in TNF-α (Zhang et al., 2018). *A. muciniphila* can also secrete a GLP-1-inducing protein (P9) that interacts with intercellular adhesion molecule 2 (ICAM-2) and promotes GLP-1 secretion, thus maintaining glucose homeostasis and ameliorating metabolic diseases in mice (Yoon et al., 2021). GLP-1 is secreted from intestinal L-cells and plays an important role in lowering blood glucose, suppressing β-cell apoptosis, and triggering β-cell replication, differentiation, and neogenesis via the enteroinsular axis (Holst et al., 2016). These data suggested that *A. muciniphila* and its byproducts may be potential drugs for improving glucose homeostasis and insulin sensitivity, restoring energy homeostasis, inhibiting low-grade inflammation, and reducing the risk of complications, such as cardiovascular disease. However, it is still unclear for the underlying mechanisms, especially their effects in human beings.

Although obesity occurs in most of T2D patients, some are underweight or have normal weight. Chenodeoxycholic acid (CDCA) is a bile acid synthesized from cholesterol in the liver, and gut microbes modulate bile acids and thereby influence host metabolism (Sayin et al., 2013). T2D patients showed alterations of BAs composition and levels in plasma (Wewalka et al., 2014; Yu et al., 2015). FGF19 is produced in the intestine, it stimulates glycogen synthesis and suppresses gluconeogenesis through insulin-independent pathways (Morton et al., 2013). FGF19 is regulated by BAs (Potthoff et al., 2012; Thomas et al., 2008). Zhang et al. showed that lean T2D patients had depleted *A. muciniphila* proportion in the gut, which was accompanied by a decrease in FGF19 expression and insulin secretion. Oral *A. muciniphila* administration improved diet-induced glucose intolerance. The mechanism underlying its beneficial effect on host metabolism relies on the downregulation of 𝛽-CDCA levels, which triggered insulin secretion and increased FGF19 expression, thus reducing blood glucose levels and restoring glucose homeostasis (Zhang et al., 2021). Therefore, *A. muciniphila* modulated the glucose homeostasis in lean T2D patients through regulating BAs, such as 𝛽-CDCA levels.

### Type 1 diabetes (T1D)

Apart from T2D, patients with autoimmune T1D also harbor different intestinal microbiota from healthy subjects (Yurkovetskiy et al., 2015); however, its causality and the role of individual microbiota members has not been conducted. *A. muciniphila* was correlated with a reduced risk of suffering from T1D-associated autoantibodies in children, indicating that this symbiont exerts an immunoregulatory role in the development of T1D (Brown et al., 2011; Endesfelder et al., 2016). Hänninen et al. found a negative correlation between *A. muciniphila* abundance and the risk of developing T1D-related autoantibodies in the NOD mouse model of T1D. *A. muciniphila* transfer increased expression of the antimicrobial peptide Reg3γ and mucus production, lowered serum endotoxin levels, islet TLR expression and TLR2/4 expression, increased Foxp3 + regulatory T cells in islets, and TGF-β and IL-10 in pancreatic lymph nodes, thus modulating immune responses and ameliorating T1D development (Hänninen et al., 2018). Further research on the potency of pasteurized or live *A. muciniphila*, *A. muciniphila*-derived products in protecting against the development of T1D is warranted, and the underlying mechanisms remain an important and largely unexplored research question.

In addition, Cold exposure markedly reshapes the gut microbial community, giving rise to a so-called “cold microbiota” in which *A. muciniphila* is among the most strongly shifted bacteria and shows a significant reduction in abundance (Chevalier et al., 2015). Cold microbiota transplantation could increase insulin sensitivity and promote cold tolerance, energy expenditure, and the intestinal absorptive surface. Moreover, *A. muciniphila* re-colonization could reduce the OGTT peak and intestinal absorptive capacity, reverse the reduction in apoptotic cells, and reduce glucose uptake genes during cold exposure (Chevalier et al., 2015).

These data implied that this bacterium may be served as an energy sensor that exhibits high level during caloric deficiency and low level during excess energy. The action of *A. muciniphila* could be a co-evolutionary mechanism promoting energy uptake when available. The above-discussed effects of *A. muciniphila* in obesity, T2D and T1D could provide a potential explanation for its roles in modulating energy metabolism (Figure 5). Although observational findings in humans and animal studies demonstrated *A. muciniphila* and its derivatives as a potential probiotic and therapies in metabolic diseases, more clinical studies are needed to substantiate the benefits of *A. muciniphila*’s supplementation. Moreover, the barriers involving large scale clinical studies, regulation and production process must be carefully inspected and resolved before they are approved for clinical application, and these currently limit clinical translation.

**Figure 5:**
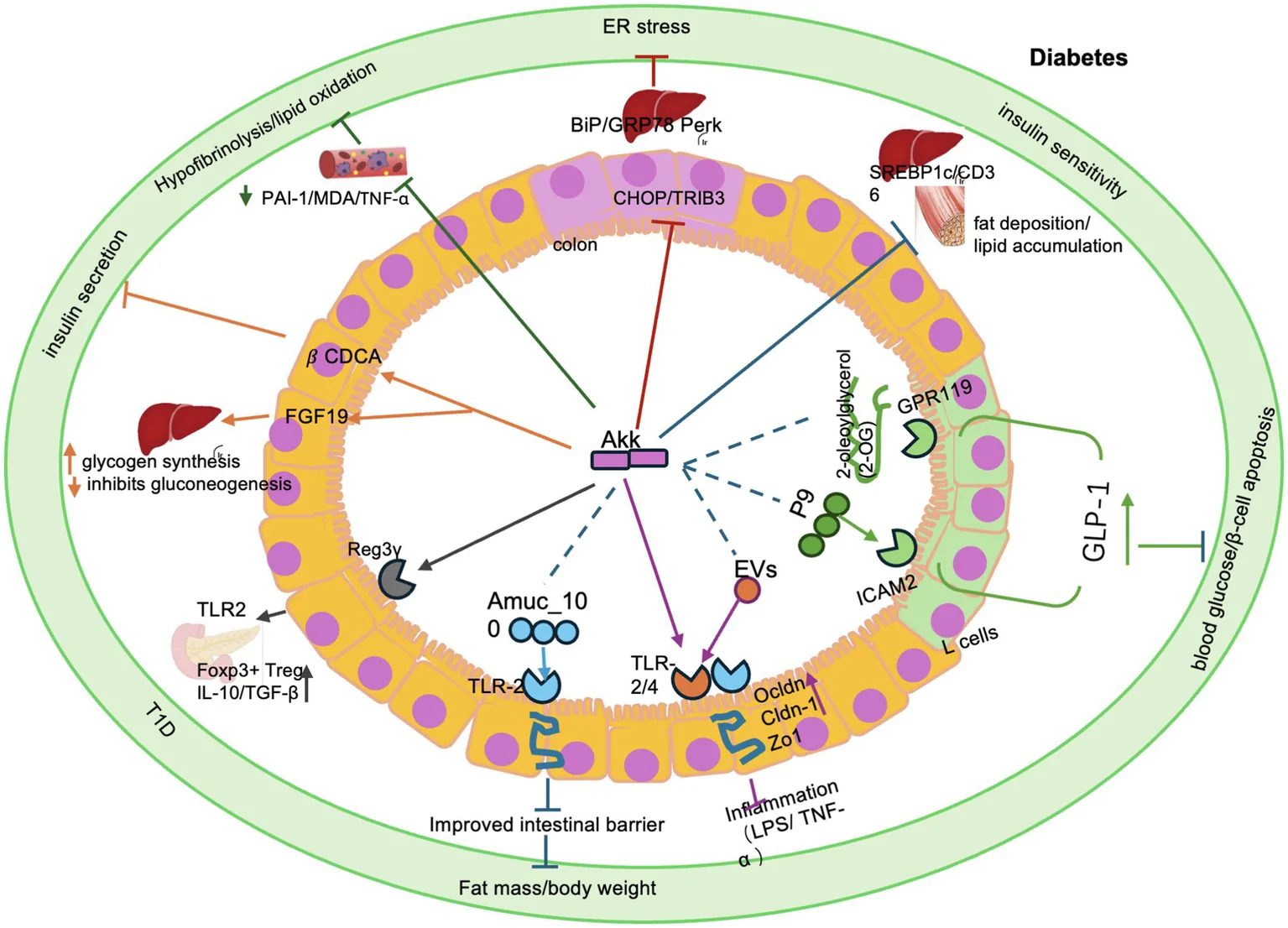
Effects of *A. mucin iphila* and its derived parts on metabolic diseases.

### Friend or foe?

As a gut commensal, *A. muciniphila* is not pathogenic to the host itself, it can be pathogenic depending on the existence of certain pathogenic bacteria and the immunological settings. Ganesh et al. showed that *A. muciniphila* intensifies gut inflammation in *Salmonella enterica serovar Typhimurium-*infected mice (Ganesh et al., 2013). Considering the reason behind this, it’s worth noting that *A. muciniphila* administration to *Salmonella enterica serovar Typhimurium*-infected mice showed decreased mucin-filled goblet cells and thinner mucus layers, which are more susceptible to pathogen exposure in the host. In addition, the existing pathogenic bacterium could be another explanation. Interactions between molecules released by specific microbes and *A. muciniphila* may also modulate its protective function and cause it to become pathogenic. In fact, it has clarified that beneficial microbes may switch to be potentially harmful for the host in pathogenic contexts in which intestinal homeostasis is disrupted (Ayres et al., 2012).

Seregin et al. demonstrated that oral administration of *A. muciniphila* worsened colitis in germ-free IL10−/− mice, suggesting that *A. muciniphila* can act as a pathway to facilitate the development of colitis in a genetically susceptible host (Seregin et al., 2017). In the same immunological settings, Ganesh et al. found that *enterococcus faecium* NCIMB 10415, a potential probiotic, does not protect IL10−/− mice from chronic gut inflammation (Ganesh et al., 2012). Moreover, they found that the low abundance of *A. muciniphila* was associated with a significantly decreased caecal inflammation score and lightened paracellular permeability (Ganesh et al., 2012). Therefore, the type of mouse with specific immunological background is also an important factor determining the effect of *A. muciniphila* on the host.

Therefore, the beneficial and the harmful roles of *A. muciniphila* in a range of immunological settings and under different host pathogenic contexts and its influencing factors deserve further investigation.

### Final marks and future perspectives

In conclusion, we reviewed the genotypic diversity of *A. muciniphila* and summarized the factors influencing its growth, such as medications, diet, lifestyle, host-derived substances, and microbial interactions. As one of the most abundant bacteria in the gut, *A. muciniphila* has sparked excitement as a promising therapeutic probiotic because of its favorable effects on human diseases, such as IBD, CDI, respiratory diseases, dermal diseases, atopic diseases, rheumatic diseases, liver diseases, metabolic diseases, and neurological disorders. Among all diseases, the role of *A. muciniphila* in metabolic diseases and the underlying mechanisms have been widely identified. Moreover, components such as EVs, Amuc_1100, and P9 proteins were demonstrated to induce beneficial M2 macrophages, reduce CTLs, suppress inflammation, improve gut barrier disturbance, reduce body weight gain, and improve glucose tolerance.

Nevertheless, the mechanisms by which *A. muciniphila* influences host metabolism remain incompletely understood, and contradictory results regarding its beneficial effects call for further research into the safety, value, and efficacy of its use as a therapy. To date, the pro-inflammatory effects observed to date are likely attributable to strain specificity, the coexistence of other pathogenic bacteria, and differences among animal models. In the future, *A. muciniphila* is expected to be developed as a next-generation probiotic, and its components hold promise as therapeutic agents.
